# Multi-omics Integrative Analysis for Incomplete Data Using Weighted *p*-Value Adjustment Approaches

**DOI:** 10.1007/s13253-024-00603-3

**Published:** 2024-02-28

**Authors:** Wenda Zhang, Zichen Ma, Yen-Yi Ho, Shuyi Yang, Joshua Habiger, Hsin-Hsiung Huang, Yufei Huang

**Affiliations:** 1 Walmart Global Tech, Sunnyvale, CA 94086 USA; 2 Department of Mathematics, Colgate University, Hamilton, NY 13346 USA; 3 Department of Statistics, University of South Carolina, Columbia, SC 29208 USA; 4 Department of Statistics, Oklahoma State University, Stillwater, OK 74078 USA; 5 Department of Statistics and Data Science, University of Central Florida, Orlando, FL 32816 USA; 6 Division of Hematology/Oncology, Department of Medicine, University of Pittsburgh, Pittsburgh, PA 15232 USA

**Keywords:** Weighted *p*-value adjustment, Missing value, Incomplete Data, Integrative multi-omics analysis, Omnibus test

## Abstract

The advancements in high-throughput technologies provide exciting opportunities to obtain multi-omics data from the same individuals in a biomedical study, and joint analyses of data from multiple sources offer many benefits. However, the occurrence of missing values is an inevitable issue in multi-omics data because measurements such as mRNA gene expression levels often require invasive tissue sampling from patients. Common approaches for addressing missing measurements include analyses based on observations with complete data or multiple imputation methods. In this paper, we propose a novel integrative multi-omics analytical framework based on *p*-value weight adjustment in order to incorporate observations with incomplete data into the analysis. By splitting the data into a complete set with full information and an incomplete set with missing measurements, we introduce mechanisms to derive weights and weight-adjusted *p*-values from the two sets. Through simulation analyses, we demonstrate that the proposed framework achieves considerable statistical power gains compared to a complete case analysis or multiple imputation approaches. We illustrate the implementation of our proposed framework in a study of preterm infant birth weights by a joint analysis of DNA methylation, mRNA, and the phenotypic outcome. Supplementary materials accompanying this paper appear online.

**Supplementary Information:**

The online version contains supplementary material available at 10.1007/s13253-024-00603-3.

## Introduction

Advancements in high-throughput technologies have enabled the generation of large-scale multi-omics data from multiple sources. Increasingly, multi-omics data such as DNA sequences, copy number variations, methylation, miRNA, and gene expression are collected from the same individuals in biomedical studies. The benefits of combining multiple data sources and performing joint analyses with all available genomic information and the phenotypic outcome are multi-fold. First, different data types could reflect various aspects of the underlying biological system (Song et al. 2020; Kristensen et al. 2014). Second, if multiple data sources all pinpoint the same gene or pathway, then it is less likely to be a false positive. Third, combining data from various sources can lead to better statistical performance in detecting signals among the noise.

**Fig. 1:**
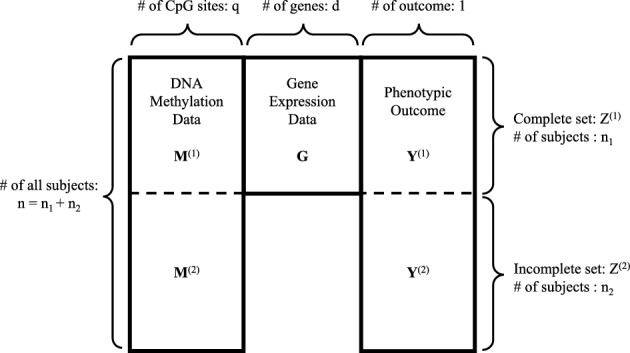
Diagram of the matrix form of the full data with complete DNA methylation ${\textbf{M}}$ but incomplete gene expression ${\textbf{G}}$

In integrative multi-omics data analysis, mRNA gene expression often serves as the intermediate variable in many underlying etiological mechanisms. Due to the fact that mRNA measurements often require invasive tissue sampling from participants, it is common to have a large portion of missing values in mRNA gene expression measurements as shown in Fig. 1. A straightforward approach for handling missing values is to implement a complete case analysis by removing observations with incomplete information (Guillermo et al. 2021; Ramaswami et al. 2020; de Silva and Perera 2017). Another solution could be to apply imputation methods Lin et al. 2016; Rubin 2004; Van Buuren and Groothuis-Oudshoorn 2011; Troyanskaya et al. 2001; Shah et al. 2014. Multiple imputation (Rubin 2004) is a widely used solution to the missing value problem. Multivariate imputation by chained equations (MICE) (Van Buuren and Groothuis-Oudshoorn 2011) is a useful tool for implementing multiple imputation to iteratively generate missing values from conditional distributions on the basis of the observed data while considering the relationships between variables.

Imputation algorithms can provide adaptable solutions for dealing with missing information. However, in the situation where there is a large proportion of missing values, imputation approaches might not perform well (Yang et al. 2014; Yu et al. 2020). Multiple imputation algorithms such as MICE can quickly become computationally intensive as the number of variables with missing values increases (Ratolojanahary et al. 2019). Furthermore, imputation methods mainly use information from single omics data rather than considering the connections among multi-omics data, which can lead to biases in the final imputation (Lin et al. 2016).

In this paper, we propose a novel integrative analytical framework using weighted *p*-value adjustment approaches to incorporate both the complete and incomplete (with missing mRNA gene expression measurements) observations in multi-omics analyses. The weighted *p*-value adjustment approaches were proposed in the context of multiple hypothesis testing to incorporate external information or prior knowledge while maintaining the type I error rate (Roeder and Wasserman 2009). Several weighting procedures have been proposed in the literature, such as weighted Bonferroni method for family-wise error rate (FWER) control (Roeder and Wasserman 2009; Li et al. 2013), weighted Benjamini–Hochberg (BH) method (Genovese et al. 2006; Habiger 2017) and *q*-value method (Storey and Tibshirani 2003; Storey et al. 2004) for false discovery rate (FDR) control, and grouped FDR methods (Ignatiadis and Huber 2021; Roquain and Van De Wiel 2009). To ensure the independence between *p*-values and the derived weights (Roeder and Wasserman 2009), the sample splitting strategy (Rubin et al. 2006; Roeder et al. 2007) provides a useful tool that uses a subset of the data to generate weights and the remaining data to compute *p*-values.

In our proposed approaches, we split the samples into a complete set with full information and an incomplete set with missing mRNA gene expression measurements. Two weighted *p*-value mechanisms (general and reverse weighting schemes) are proposed. Compared to integrative procedures that utilize Markov chain Monte Carlo such as iBAG (Wang et al. 2013), Bayesian integrative model (Fridley et al. 2012), multi-dataset integration (Kirk et al. 2012), and Bayesian consensus clustering (Lock and Dunson 2013), our proposed approach is fast and computationally simple for a whole-genome study. Computational efficiency is particularly critical for integrating multi-omics data since the interactions between multiple data types grow exponentially with the number of variables considered in the study.

In this paper, we describe the proposed weighted *p*-value mechanisms in Sect. 2. We demonstrate the advantages of our proposed approach compared to imputation algorithms in simulation studies in Sect. 3. To illustrate the use of our proposed approaches, we apply them to jointly analyze DNA methylation, gene expression, and phenotypic outcome in a preterm infant birth weights study in Sect.  4. Finally, we conclude with a discussion in Sect. 5.

## Materials and Methods

### Datasets and Databases

The dataset considered in this paper came from a genetic association study for preterm infants (Kashima et al. 2021) and can be accessed through Gene Expression Omnibus (GEO) with accession number GSE110828. This study contains 157 observations with DNA methylation and phenotypic outcome information. However, mRNA gene expression measurements were collected for only 55 observations (65% missing). DNA methylation levels were measured using the Ilumina HumanMethylation450 BeadChip for 410,735 cytosine–phosphate–guanine (CpG) sites and reported after quantile normalization and background correction. The mRNA gene expression levels of 46,789 transcripts were profiled using the SurePrint G3 Human GE microarray 8$\times$60K version 3.0 (Agilent Technologies). Transcriptional activities were analyzed using GeneSpring 14.5 to perform probe filtering and quantile normalization to report the gene expression signal levels.

### Models

Let ${\textbf{Y}}=(Y_1,\ldots ,Y_n)^T$ be the vector of phenotypic outcome with *n* representing the total number of subjects, ${\textbf{X}}$ be the matrix of clinical covariates, and ${\textbf{M}}=({\textbf{M}}_{1}, \ldots , {\textbf{M}}_{q})$ be the matrix of DNA methylation levels of *q* CpG sites, where ${\textbf{M}}_j=(M_{1j},\ldots ,M_{nj})^T$, $j=1,\ldots ,q$, is the vector of methylation levels for the *j*th CpG site. Let ${\textbf{G}}=({\textbf{G}}_{1},\ldots ,{\textbf{G}}_{d})$ be the matrix of standardized mRNA gene expression data (mean = 0 and standard deviation = 1) of *d* genes and ${\textbf{G}}_l=(G_{1l},\ldots ,G_{n_1l})^T$ be the vector of expression levels for the *l*th gene ($l=1,\ldots ,d$) with $n_1$ representing the number of subjects of gene expression data ($n_1 \le n$). All subjects can be split into two subsets: a complete set ($Z^{(1)}=({\textbf{M}}^{(1)},{\textbf{Y}}^{(1)}, {\textbf{X}}^{(1)}, {\textbf{G}})$) with $n_1$ subjects where mRNA expression data can be observed and an incomplete set ($Z^{(2)}=({\textbf{M}}^{(2)},{\textbf{Y}}^{(2)}, {\textbf{X}}^{(2)})$) with $n_2$ subjects where the mRNA gene expression data are completely missing. The total number of subjects is $n=n_1+n_2$. Note that in some situations, the covariates ${\textbf{X}}$ may not be included in the study. In this case, the complete set is $Z^{(1)}=({\textbf{M}}^{(1)},{\textbf{Y}}^{(1)}, {\textbf{G}})$ and the incomplete set is $Z^{(2)}=({\textbf{M}}^{(2)},{\textbf{Y}}^{(2)})$. Figure 1 provides a diagram of the matrix form of the data without the covariates ${\textbf{X}}$. A diagram illustrating the data collection process is shown in Web Fig. 1 in Web Appendix A.

#### General Weight

In the complete set, $Z^{(1)}$, we consider the integrative analytical framework (IG) proposed by Zhao et al. (2014) to integrate the DNA methylation, mRNA gene expression data, and the phenotypic outcome to derive the *p*-value ($p_j^{IG}$) for testing the association between the *j*th DNA methylation measurement ($j=1,\ldots ,q$) and the phenotypic outcome. Other integrative analysis approaches can also be used instead. A detailed description of the IG approach can be found in the paper authored by Zhao et al. (2014). Briefly, $p_j^{IG}$ is calculated via two linear models formulated as follows:

1 $$\begin{aligned} E(Y_i^{(1)}| {\textbf{G}}_i,{\textbf{X}}^{(1)}_i)= & {} \alpha _0 + {\textbf{G}}_i^T\mathbf {\alpha _G} + ({\textbf{X}}_i^{(1)})^T\mathbf {\alpha _X} \end{aligned}$$

2 $$\begin{aligned} {\textbf{G}}_i^T\mathbf {\alpha _G}= & {} \beta _{0j} + \beta _{M_j} M_{ij}^{(1)} + ({\textbf{X}}^{(1)}_i)^T\mathbf {\beta _X} + u_{ij}, \end{aligned}$$

where $\alpha _0$ and $\beta _{0j}$ are the intercepts and $\mathbf {\alpha _G}$ is the coefficient describing the association between mRNA gene expression and the outcome. The clinical covariates ${\textbf{X}}$ may have a direct effect on the outcome through the coefficient $\mathbf {\alpha _{X}}$ or an indirect effect via its association with the gene expression ${\textbf{G}}$ through the coefficient $\mathbf {\beta _{X}}$. Hence, they are included in both Equation (1) and (2). The parameter of interest, $\beta _{M_j}$, measures the association between the *j*th DNA methylation level and the phenotypic outcome via the regulation of mRNAs; and $u_{ij}\sim {\mathcal {N}}(0, \sigma ^2_{u})$ is the error term ($i=1,\ldots ,n_1$) with variance $\sigma ^2_{u}$. Let $\widehat{\mathbf {\alpha _G}}$ and $\widehat{\beta _{M_j}}$ be the estimates of $\alpha _{G}$ and $\beta _{M_j}$. In practice, $\widehat{\beta _{M_j}}$ can be estimated via Equation (2) with $\widehat{\mathbf {\alpha _G}}$ derived from Equation (1). Under the null hypothesis of no association between $M_j$ and *Y* ($H_0:\beta _{M_j}=0$), the *p*-value for $\beta _{M_j}$ is calculated as follows. First, a linear model for the complete data in Equation (1) is fitted to obtain the estimate ${\widehat{\alpha }}_G$. The estimate ${\widehat{\alpha }}_G$ is used to compute ${\textbf{G}}_i^T{\widehat{\alpha }}_G$, which is then regressed onto DNA methylation $M_{ij}$ and covariates ${\textbf{X}}_i$ in Equation (2) to obtain ${\widehat{\beta }}_{M_j}$, the regression coefficient of DNA methylation at the $j^{th}$ CpG site. The *p*-value for testing the association of the $j^{th}$ CpG site is obtained from the *t*-statistic

$$\begin{aligned} \frac{{\widehat{\beta }}_{M_j} - 0}{se_{{\widehat{\beta }}_{M_j}}} \end{aligned}$$

for $H_0:\beta _{M_j}=0$ versus $H_1:\beta _{M_j}\ne 0$, where $se_{{\widehat{\beta }}_{M_j}}$ is the standard error of ${\widehat{\beta }}_{M_j}$.

In the incomplete set, $Z^{(2)}$, we implement the linear model

3 $$\begin{aligned} Y_i^{(2)} = \gamma _{0j} + \gamma _{M_j}M_{ij}^{(2)} + ({\textbf{X}}_i^{(2)})^T \mathbf {\gamma _X} + \epsilon _{ij}, \end{aligned}$$

where $\epsilon _{ij}\overset{iid}{\sim }{\mathcal {N}}(0, \sigma ^2_{\epsilon })$, $i = 1,\ldots ,n_2$ and $j=1,\ldots ,q$, is the error term with variance $\sigma ^2_{\epsilon }$; $\gamma _{0j}$ is the intercept and $\gamma _{M_j}$ represents the association between *j*th methylation data measurement and the phenotypic outcome; and $\mathbf {\gamma _X}$ is the vector of coefficients for the covariates. Let $\widehat{\gamma _{M_j}}$ be the estimate of $\gamma _{M_j}$. The *p*-value ($p^{LM}_j$) can be derived based on $\widehat{\gamma _{M_j}}$ and var($\widehat{\gamma _{M_j}}$) under the null hypothesis, $\gamma _{M_j}=0$. As described in Zhao et al. (2014), when Equation (1) and  (2) hold, we can plug Equation (2) into Equation (1), and hence, testing the null hypothesis of $\gamma _{M_j}=0$ in equation (3) is equivalent of testing that of $\beta _{M_j}=0$ in Equation (2).

In the general weighting scheme, $p^{LM}_j$ derived from $Z^{(2)}$ is used to generate the weight. As suggested by Li et al. (2013), we set $w_{G_j}= \sqrt{-\log _{10} (p^{LM}_j)}$ when $p^{LM}_j < 0.05$, and set $w_{G_j}=1$ otherwise. Since smaller *p*-values are associated with null hypotheses that are more likely to be false, the proposed weights are anticipated to be positively correlated with optimal weights and perform well (Habiger 2017). To control the type I error, the general weights are then divided by the average weight $w^*_{G_j}=w_{G_j}/\overline{w_G}$ ($\overline{w_G}=1/q\sum _{j=1}^q w_{G_j}$) to ensure $\overline{w^{*}_{G}} = 1$ (Genovese et al. 2006; Wasserman and Roeder 2006D). Finally, the adjusted *p*-values for general weighting scheme can be calculated as $p_{1j}=p^{IG}_j/w^*_{G_j}$.

#### Reverse Weight

The general weighting scheme is more effective when the missing rate of gene expression data is low. When the missing rate is high (i.e., $>50\%$), we propose a reverse weighting scheme to increase the power of identifying significant CpG sites. This approach to deriving weights by a reverse weighting scheme is similar to the general weighting scheme but uses $Z^{(1)}$ to obtain weights while deriving *p*-values using $Z^{(2)}$.

In $Z^{(2)}$, the weights are calculated in terms of $p_j^{IG}$ obtained from $Z^{(1)}$ by implementing the IG model. The reverse weight is set to be $w_{R_j}=\sqrt{-\log _{10} (p^{IG}_j)}$ when $p^{IG}_j < 0.05$ and $w_{R_j}=1$ otherwise. Then the weights are adjusted by the average value as follows, $w_{R_j}^*=w_{R_j}/\overline{w_{R}}$, where $\overline{w_R}=1/q\sum _{j=1}^q w_{R_j}$, to ensure $\overline{w^{*}_{R}}=1$ (Genovese et al. 2006; Wasserman and Roeder 2006D). Finally, we derive the *p*-value adjusted by the corresponding reverse weight, $p_{2j}=p^{LM}_j/w^*_{R_j}$.

#### Omnibus Method

For the *j*th CpG site ($j=1, 2,\ldots , q)$, we obtain adjusted p-values, $p_{1j}$ and $p_{2j}$ from the general and the reverse weighting scheme, respectively. We consider an omnibus approach, the aggregated Cauchy association test (ACAT) (Liu et al. 2019; Liu and Xie 2020), to combine the two adjusted *p*-values. The ACAT calculates the test statistic via a weighted sum of Cauchy transformations of the component *p*-values:

4 $$\begin{aligned} T^{ACAT}_j= (1-\lambda ) \times tan\{(0.5-p_{1j})\pi \} + \lambda \times tan\{(0.5-p_{2j})\pi \}. \end{aligned}$$

In equation 4, $p_{1j}$ from the general and $p_{2j}$ from the reverse weighting scheme are combined for the *j*th CpG site and $\lambda$ is $0\le \lambda \le 1$. Because the general scheme is more powerful when the missing rate is low, while the reverse scheme becomes more effective when the missing rate is greater than $50\%$, we set $\lambda$ as the missing proportion of the gene expression data. Therefore, the adjusted *p*-values from the general weighting scheme are emphasized in studies with low missing rates and vice versa.

## Simulation

### Settings

We conducted simulation studies to compare the performance of the proposed weighting approaches to the IG method (Zhao et al. 2014), the popular MICE imputation (Van Buuren and Groothuis-Oudshoorn 2011), and the K-nearest-neighbor (KNN) imputation method (Batista and Monard 2002) under various scenarios. In this section, we use the notation $\gamma _{MG}$ to describe the DNA–gene association between DNA methylation loci and gene expressions, and $\gamma _{GY}$ to denote the association between gene expressions and the phenotypic outcome. Since there were 157 observed subjects in the experimental dataset, we generated $n=150$ samples in all scenarios and studied the power of models averaged over 1,000 simulation iterations.

The following steps describe the data generation procedures for Scenarios I and II with low-dimensional gene expression data. For the *i*th subject, we first generated data for $q=5$ DNA methylation loci (${\textbf{M}}_i$) and $r=2$ clinical covariates (${\textbf{X}}_i$) from standard normal distributions. A single CpG site was selected to be the true underlying methylated CpG site associated with the phenotypic outcome. We denote the methylation of this CpG site by $M_{i1}$. A related simulation scenario where the phenotypic outcome is associated with multiple CpG sites is presented in detail in Wed Appendix B. The results are similar to the results presented here.

Then, we considered $d=8$ genes with expression levels (${\textbf{G}}_i$), of which 3 genes were simulated based on the underlying CpG site ($M_{i1}$) via the linear model,

5 $$\begin{aligned} {\textbf{G}}_i = \mathbf {\gamma }_{0G} + M_{i1} \mathbf {\gamma }_{MG} + {\textbf{X}}_i^T \mathbf {\gamma }_{XG} + \mathbf {\epsilon }_{i1}, \end{aligned}$$

where $\mathbf {\gamma }_{MG}$ is the vector of DNA–gene association; and $\mathbf {\epsilon }_{i1} \sim \mathbf {{\mathcal {N}}}({\textbf{0}}, {\textbf{I}})$ and ${\textbf{I}}$ is an identity matrix. The values of the intercept ($\mathbf {\gamma }_{0G}$) were determined based on the mean expression levels of randomly selected genes from the experimental dataset. The values of the elements in the coefficient vectors ($\mathbf {\gamma }_{XG}$) were all set equal to 0.5. The five other genes served as unrelated signals and were generated from the normal distribution, ${\mathcal {N}}(\mu _{0G}, 1)$, where $\mu _{0G}$ was also determined by the mean expression level of a randomly picked gene from the experimental dataset described in Sect. 4.

Finally, we simulated $Y_i$ based on the mRNA expression levels of the three modulating genes, according to the second linear model,

6 $$\begin{aligned} Y_i = \gamma _{0Y} + {\textbf{G}}_i^{T} \mathbf {\gamma }_{GY} + {\textbf{X}}_i^T \mathbf {\gamma }_{XY} + \epsilon _{i2}, \end{aligned}$$

where $\epsilon _{i2}\sim {\mathcal {N}}(0, 1)$ is the error term with the variance of the phenotypic outcome being set equal to 1. Here, the intercept ($\gamma _{0Y}$) was set equal to the mean birth weight score of the preterm infants in the experimental dataset, and the associations between the clinical covariates and the phenotypic outcome ($\mathbf {\gamma }_{XY}$) were all set equal to 0.5. Without loss of generality, we also assumed the same values for all the elements in the vector $\mathbf {\gamma }_{GY}$. After generating $n=150$ subjects, which is close to the sample size of the experimental dataset, multiple records of gene expression levels were removed randomly.

#### Scenario I

In Scenario I, we set both $\gamma _{MG}$ and $\gamma _{GY}$ equal to 0. The missing proportion was set to be 20%, 50%, and 70%. After obtaining the weight-adjusted *p*-values via the proposed weighting schemes, we considered both the weighted Bonferroni method for FWER control (Bland and Altman 1995) and the *q*-value method (Storey and Tibshirani 2003; Storey 2003; Storey et al. 2004) for FDR control. The FWER was calculated as the proportion of times that at least one significant CpG site was observed among all CpG sites. The FDR was calculated as the ratio of falsely detected CpG sites after the *q*-value adjustment. The results for FWER and FDR at the nominal level of 0.05 are reported in Table 1.

#### Scenario II

In this scenario, we examine the power of the proposed methods for identifying $M_1$ under varying missing proportions in gene expression data and various combinations of DNA–gene and gene–phenotype associations ($\gamma _{MG}$ was set equal to 0, 0.1, 0.2, and 0.5, and $\gamma _{GY}$ was set equal to 0.1 and 0.2.) We calculate power of detecting $M_1$ (the true DNA methylation signal) over 1,000 simulation iterations.

#### Scenario III

In this scenario, we considered 1,000 mRNA expression measurements and assumed that $M_{i1}$ was associated with $Y_i$ for the *i*th subject through the regulation of $k=5$ genetic pathways (${\textbf{f}}_i$). The associations between ${\textbf{G}}_i$ and ${\textbf{f}}_i$ could be estimated using a factor model (Baek et al. 2020),

7 $$\begin{aligned} {\textbf{G}}_i = {\textbf{B}}{\textbf{f}}_i + {\textbf{U}}_i, \end{aligned}$$

where ${\textbf{f}}_i\in {\mathcal {R}}^{k}$ ($k<d$) is the vector of latent factors with $cov({\textbf{f}}_i)={\textbf{I}}_k$, ${\textbf{U}}_i\in {\mathcal {R}}^{d}$ is the error term, and $B \in {\mathcal {R}}^{d\times k}$ is the loading matrix describing the gene–factor associations. Here, *d* is the number of mRNAs (*d*=1,000 in this simulation scenario).

For *i*th subject, five factors were first simulated based on $M_{i1}$ from the equation,

8 $$\begin{aligned} {\textbf{f}}_i = M_{i1} \mathbf {\gamma }_{MG} + {\textbf{X}}_i^T \mathbf {\gamma }_{XG} + \mathbf {\epsilon }_{i1}, \end{aligned}$$

where $\mathbf {\epsilon }_{i1} \sim \mathbf {{\mathcal {N}}}({\textbf{0}}, {\textbf{I}})$, and $\mathbf {\gamma }_{XG}$ were set equal to 0.5. Second, $Y_i$ was generated based on the ${\textbf{f}}_i$ from the equation,

9 $$\begin{aligned} Y_i = \gamma _{0Y} + {\textbf{f}}_i^{T} \mathbf {\gamma }_{GY} + {\textbf{X}}_i^T \mathbf {\gamma }_{XY} + \epsilon _{i2}, \end{aligned}$$

where $\epsilon _{i2} \sim {\mathcal {N}}(0, 1)$ is the error term, and $\mathbf {\gamma }_{XY}$ and $\gamma _{0Y}$ were set to the same values as the low-dimensional cases.

In the last step, ${\textbf{G}}_i$ were generated based on ${\textbf{f}}_i$, ${\textbf{U}}_i\sim \mathbf {{\mathcal {N}}}({\textbf{0}}, {\textbf{I}})$, and ${\textbf{B}}$ Baek et al. (2020). We formed ${\textbf{B}} = 1 / \sqrt{n} \times {\textbf{L}}^T{\textbf{E}}$ where ${\textbf{L}}\sim {\mathcal {N}}({\textbf{0}}, {\textbf{I}})$ and ${\textbf{E}}$ is an $n\times k$ orthogonal matrix formulated by the eigenvectors corresponding to the *k* largest eigenvalues of ${\textbf{L}}{\textbf{L}}^T$. The optimal *k* can be estimated by minimizing the cross-validated mean squared error (MSE) (Owen and Perry 2009).

We performed the singular value decomposition (SVD) using the gene expression matrix ${\textbf{G}}$ to identify the latent pathways corresponding to the *k* largest eigenvalues. These latent pathways are then used in Equations (1) and (2) in place of ${\textbf{G}}_i$ to derive $p_j^{IG}$ (reducing dimension from *d* to *k*).

Finally, we reported the empirical power of the underlying CpG site with methylation for $\gamma _{MG}$ ranging from 0 to 0.5. The gene–phenotype association $\gamma _{GY}$ was set equal to 0.1 and 0.2, and the missing rate was 70%. Importantly, multiple imputation is computationally infeasible in this high-dimensional scenario.

#### Competing Methods

Four competing methods were considered in the simulation studies: (1) complete case studies using only the complete set with the integrative analytical framework (IG) (Zhao et al. 2014), (2) KNN imputation algorithm (KNN impute)(Batista and Monard 2002) to estimate the missing values using the mean of the nearest values of *k*th closest subjects, (3) multivariate imputation via chained equations (MICE) method to estimate the missing values by combining results derived from multiple imputed datasets, and (4) linear model on all subjects of ${\textbf{M}}$ and ${\textbf{Y}}$. In (2) and (3), the IG model was implemented to identify the underlying CpG site after imputing the missing values in the gene expression data.

We implemented a 10 nearest-neighbor imputation method with the impute package (Hastie et al. 2001) and applied the MICE algorithm with the mice package (Van Buuren and Groothuis-Oudshoorn 2011) in R. The maximum number of iterations was set equal to 5 in MICE, and 5 datasets were generated for pooling results. Due to the intractable computational time in the high-dimensional case, MICE was not implemented in Scenario III.

### Simulation Results

Based on simulation Scenario I, Table 1 reports the FWER after the weighted Bonferroni method and the FDR after *q*-value method of testing the CpG sites with $\gamma _{MG}=\gamma _{GY}=0$ (no association with the outcome ${\textbf{Y}}$). The results show that our proposed methods and the existing method maintained both FWER and FDR at the nominal 0.05 level.

**Table 1:** Type I error control for FWER using weighted Bonferroni method and FDR using *q*-value method when $\gamma _{MG}=\gamma _{GY}=0$

				KNN	General	Reverse	Omnibus	Linear
		IG	MICE	Impute	Weight	Weight	Method	Model
20%	FWER	0.037	0.010	0.045	0.037	0.048	0.047	0.053
	FDR	0.037	0.010	0.052	0.037	0.049	0.053	0.057
50%	FWER	0.063	0.013	0.061	0.061	0.060	0.065	0.053
	FDR	0.062	0.013	0.061	0.062	0.059	0.077	0.057
70%	FWER	0.028	0.026	0.044	0.027	0.045	0.048	0.053
	FDR	0.031	0.027	0.050	0.030	0.040	0.054	0.057

Results were calculated from 1000 simulation iterations with $n=150$

**Fig. 2:**
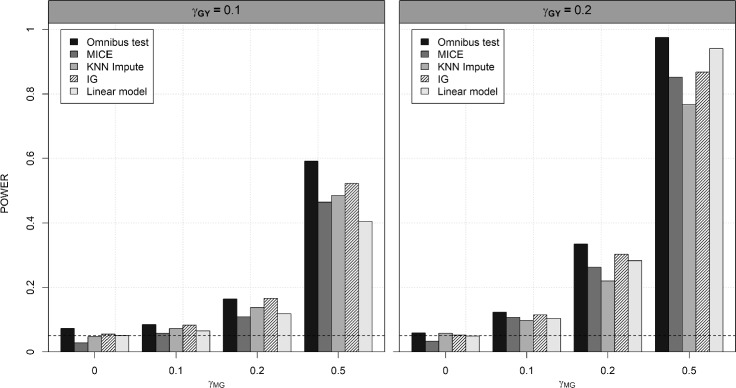
Power comparisons of the omnibus method, MICE, KNN imputation, IG, and linear model for $\gamma _{MG}$ ranging in 0, 0.1, 0.2, and 0.5. The value of $\gamma _{GY}$ was set equal to 0.1 or 0.2. The standard deviations of both $\gamma _{MG}$ and $\gamma _{GY}$ were set equal to 1. Power was calculated from 1,000 simulation iterations with $n=150$

Based on Simulation Scenario II, Fig. 2 presents the average power of the proposed omnibus method and the competing methods to compare the performance for identifying the underlying CpG site. In datasets with a high missing rate ($70\%$), the proposed omnibus method is more powerful than the IG model with the complete case analysis and the imputation algorithms. For example, when $\gamma _{MG}=\gamma _{GY}=0.2$, the proposed omnibus method achieves the highest power, which is 10.5% higher than the IG model and 52.3% higher than MICE. Furthermore, the proposed omnibus method (0.027 s) is much faster than the MICE method (1.553 s).

In Web Appendix B, Web Fig. 2 presents the performance using the general weighting scheme, the reverse weighting scheme, and the omnibus method. Based on our simulation results, the general weighting scheme performs better in dataset with a low missing rate, while the reverse weighting scheme performs better with a high missing rate ($> 50\%$). We set $\lambda$ in the ACAT test statistic as the missing rate as described in Sect. 2.2.3. Our results show that the proposed omnibus method demonstrates competitive performance under various missing rates.

**Fig. 3:**
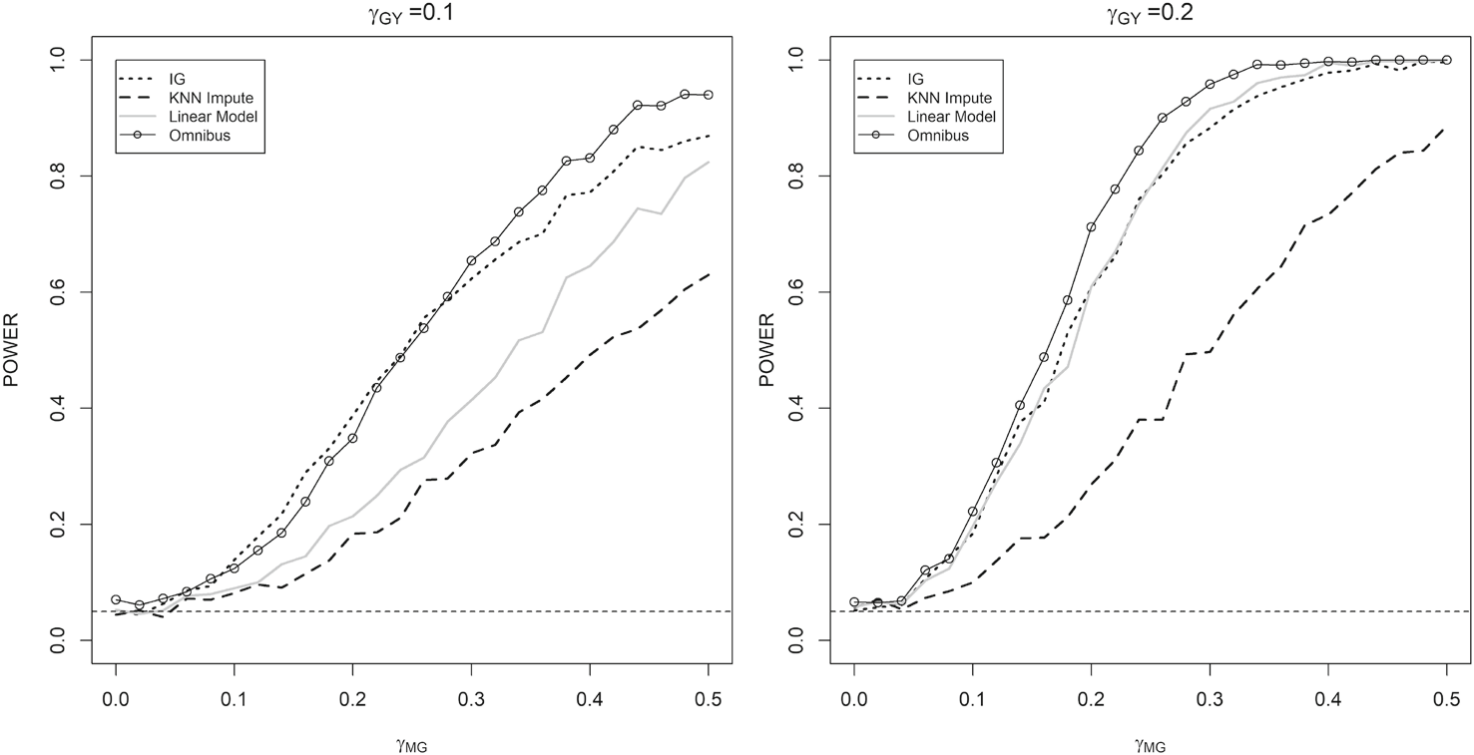
Power curves for omnibus method and competing methods in high-dimensional case based on 1,000 simulation iterations with $n=150$. The number of factors $k=5$ was determined by the tenfold cross-validation. The gene–phenotype association $\gamma _{GY}$ was set to be 0.1 or 0.2

The performance of our proposed method in a high-dimensional case is illustrated in Fig. 3. In this scenario, the missing rate was set equal to 70% to mimic that of the experimental dataset considered in this paper. As shown in the power plots, the omnibus method achieves competitive performance compared to other existing approaches. The KNN impute approach performs the worst in this setting due to the curse of dimensionality.

## Experimental Data Analysis

We implemented our proposed omnibus weighting approach using the preterm infant data (Kashima et al. 2021; Agha et al. 2016; Oken et al. 2003) described in Sect. 2.1. The infant birth weight scores were used as the phenotypic outcome. The weight scores were calculated by the normal quantile of the birth weights for each gestational age in the entire population of newborn infants so that they are normally distributed, as described in Kashima et al. (2021). The methylation levels were measured by $\beta$ values ranging from 0 (completely unmethylated) to 1 (completely methylated) to indicate the intensity of methylation on each CpG site (Kashima et al. 2021). The methylation intensities and gene expression measurements were then quantile normalized before the analysis. In addition, the birth weight scores were scaled to have a mean of 0 and a variance of 1.

The clinical covariates considered in this analysis included paternal age, maternal age, paternal body mass index (BMI), maternal BMI, maternal smoking status before pregnancy, and the gender of the infants. To correct for population stratification, we implemented the surrogate variable analysis (SVA) (Leek and Storey 2007) to account for the unobserved effect. The genomic inflation factor (van Iterson et al. 2017) was used after SVA for adjusting the inflated *p*-values due to population stratification. The quantile–quantile plot for the omnibus method presented in Web Appendix C suggests proper type I error control.

As described in Sect. 2.1, data was collected from 157 participants. However, mRNA gene expression measurements were only available for 55 participants. In the complete dataset, after normalizing the mRNA expression measurements, we implemented the dimension reduction method described in Sect. 3.1.3 for 46,789 mRNA expression measurements. The optimal number of factors was determined by minimizing the Wold-style tenfold cross-validated MSE (Owen and Perry 2009).

**Fig. 4:**
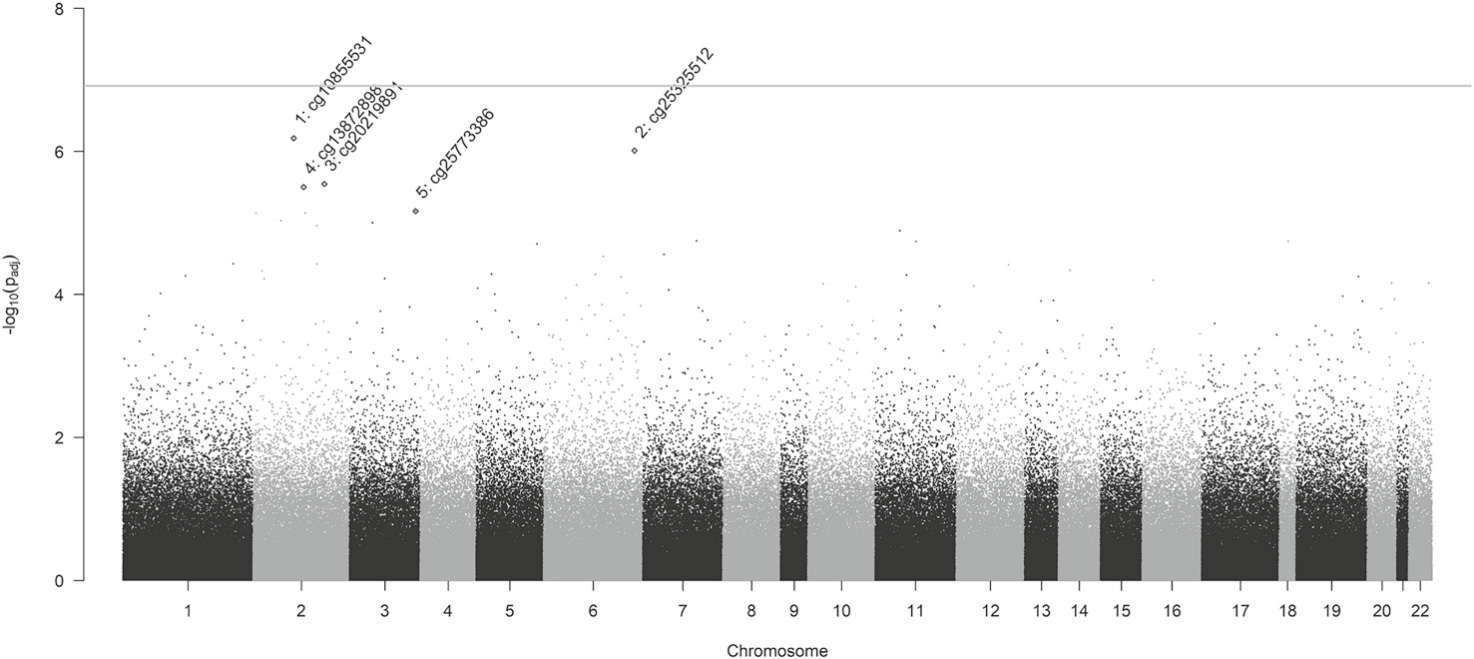
Manhattan plot for cytosine–phosphate–guanine (CpG) sites associated with preterm infants’ birth weights. The weighted *p*-values after logarithmic transformation ($-\log _{10}P_{adj}$) were used as the y-axis. The gray solid line represents the family-wise error rate (FWER) threshold under the weighted Bonferroni method. Four CpG sites with the strongest significant associations with the preterm infants’ birth weights were labeled with the corresponding CpG site IDs

For 410,735 CpG sites, we implemented our proposed weighting schemes one CpG site at a time to derive the weighted *p*-values for identifying the association with infant birth weights. The weighted *p*-values for all CpG sites with the corresponding chromosomes are presented in Fig. 4. The top 15 CpG sites are listed in Table 2 with the corresponding reference sequence (RefSeq) gene symbols. After implementing our proposed omnibus method, none of the CpG sites was identified as significant with either the *q*-value method or the weighted Bonferroni method.

**Table 2:** Top table for 15 CpG sites associated with birth weight scores of preterm infants with the smallest weighted *p*-values derived from the proposed omnibus method

Rank	Chromosome	CpG site	Gene name	Weighted *p*-value	*q*-value
1	11	cg04549076	PRG2	8.98e-07	0.295
2	4	cg14818154	ANTXR2	1.75e-06	0.295
3	20	cg26540123	WFDC3	2.16e-06	0.295
4	3	cg04127903	AHSG	8.81e-06	0.651
5	2	cg11074070	CTNNA2	1.13e-05	0.651
6	17	cg14655552	ACCN1	1.83e-05	0.651
7	12	cg17085352	HOXC13	2.10e-05	0.651
8	3	cg10805254	RYBP	2.20e-05	0.651
9	1	cg20801751	C1orf69	2.67e-05	0.651
10	17	cg07425985	ANKFY1	2.68e-05	0.651
11	17	cg24675735	MGAT5B	2.74e-05	0.651
12	8	cg22268164	TRHR	3.08e-05	0.651
13	10	cg23339629	TACR2	3.43e-05	0.651
14	7	cg10374862	MTERF	3.52e-05	0.651
15	14	cg07688213	BATF	4.38e-05	0.651

Chromosomes, CpG sites, UCSC RefSeq gene names, weighted *p*-values, and *q*-values are reported. The results were adjusted for paternal age, maternal age, paternal BMI, maternal BMI, maternal smoking status before pregnancy, and the gender of infants

**Fig. 5:**
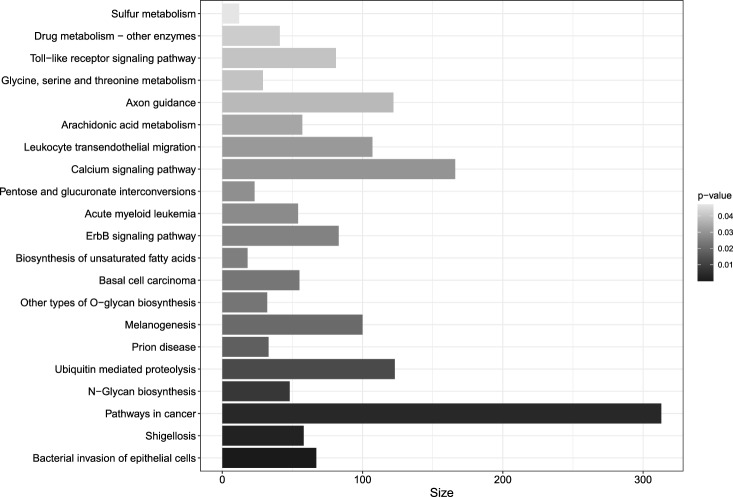
KEGG pathway enrichment analysis based on the weighted *p*-values using methylGSA Ren and Kuan (2019). Presented here are pathways with unadjusted *p*-value $<0.05$. After adjusting for multiple testing using the Benjamini–Hochberg procedure, the only FDR significant pathway is bacterial invasion of epithelial cells (*q*-value $=0.010$)

Fifteen CpG sites are listed in Table 2 in ascending order of the *p*-values derived from the omnibus method after SVA and the genomic inflation factor adjustment. In addition, based on the weighted *p*-value from all CpG sites, we performed a KEGG pathway enrichment analysis using the methylGSA algorithm in the gene set analysis for DNA methylation datasets (Ren and Kuan 2019). Figure 5 presents twenty-one KEGG pathways with unadjusted *p*-values less than 0.05. After adjusting for multiple testing using the Benjamini–Hochberg procedure, we identified one enriched pathway: bacterial invasion of epithelial cells. Pathways reported by our analysis and Kashima et al. (2021) hint at common mechanisms that are associated with low birth weights in preterm infants, such as the initiation of inflammatory responses, cytokine–cytokine receptor interaction, coregulation of ErbB, and estrogen signaling.

## Discussion

In this paper, we propose a novel framework to implement integrative analysis for multi-omics data where the intermediate variables, such as mRNA gene expression measurements, are completely missing for a large proportion of subjects. Existing multi-omics integrative studies require removing missing records or applying data imputation techniques to prepare a complete dataset for analysis. However, when the missing rate is high, especially higher than 50%, the power of complete case analysis and imputation methods decreases drastically due to the reduction in sample size. Our proposed framework utilizes a *p*-value weighted adjustment and hence incorporates information from both complete and incomplete observations in the data.

The advantages of implementing the proposed framework in the multi-omics integrative analysis are multi-fold. First, by incorporating the information from incomplete observations, our proposed approaches boost the power of multi-omics integrative analyses compared to the existing methods. Second, our proposed approaches perform well even in situations with a large missing proportion of intermediate variables. Third, the two-component weighting schemes combined in the omnibus test can provide flexibility in the implementation of multi-omics integrative analyses with missing rates ranging from 0 to 1. Furthermore, our simulation analyses showed that the proposed method maintains proper FWER control with the weighted Bonferroni method and FDR control with the *q*-value method. According to Storey and Tibshirani (2003) and Storey et al. (2004), the utilization of the *q*-value method can also maintain FDR even with weak dependence structures between CpG sites.

In our proposed method, the two-component weighting schemes perform differently in datasets with various missing rates. According to Sect. 3.2, the general weighting scheme achieves greater power in cases where the missing rates are lower than 50%, while the reverse weighting scheme achieves better performance when the missing rates are high. The proposed omnibus method is effective in that it up-weights the general scheme in situations where the missing rate is low and vice versa. The simulation studies show that the proposed omnibus method demonstrates competitive results in both low- and high-missing rate situations. Our proposed methods are best suited when there is a large proportion of missing records in the intermediate variables. In the situation when phenotypic outcomes or independent variables are also missing, methods such as data imputations could be considered in conjunction with our proposed methods.

We implemented the proposed method in a birth weight study of preterm infants and examined the CpG sites with DNA methylation that are associated with birth weights of preterm infants via the regulation of gene expression. In practice, our analytical framework can be directly applied to any continuous predictor variables (such as DNA methylation levels) or discrete variables (such as SNP genotypes or DNA mutation status). Since the measurements were generated via microarray experiments, we assumed that the intermediate variables followed normal distributions. However, if the intermediate measurements are generated by high-throughput RNA sequencing (RNAseq), preprocessing procedures such as normalization by the sequencing depth and log transformation of the data, as described in the limma (Cloonan et al. 2008) or voom (Law et al. 2014), could be applied to ensure normality before implementing the weighting schemes.

In the experimental data analysis, we considered the model proposed by pediatric scientists (Kashima et al. 2021) and did not include the interactions in this paper. However, as was shown in Zhao et al. (2014), the proposed model in Equations (1) and (2) can be readily modified to include interaction terms between gene expression, methylation, and clinical covariates. Furthermore, in the situation when clinical covariates X are not included in the study, Equations (1) and (2) could also be readily modified accordingly.

In this paper, we considered the approach proposed by Zhao et al. (2014) in our proposed methods; other integrative analysis approaches could be easily adapted to our weight adjustment framework. Compared to the two-stage approaches (the methylation model and the transcription model) implemented by Kashima et al. (2021), the proposed unified integrative analytic framework provides a straightforward way to control overall FDR at the nominal level. The main contribution of our work here is to combine the information in both complete and incomplete data through p-value weight adjustment for statistical power gain. A major benefit of this approach is the ease of computation, which is becoming increasingly important in big data analysis.

As discussed in Sect. 3.1.3, the implementation of SVD-based dimension reduction techniques allows us to apply our integrative framework to datasets with high-dimensional intermediate variables. Other variable selection approaches such as LASSO could also be used to reduce the dimension of mRNA gene expression in the analysis. Another future research area is the Cox model for survival outcomes in the integrative framework. Applying the Cox regression in our proposed integrative framework would require the implementation of estimating equation theory and to derive the asymptotic distribution of the estimates (Zhao et al. 2014). Therefore, further work is needed to develop multi-omics integration frameworks for survival outcomes.

## Supporting Information

Web Appendices and figures referenced in this paper are included as online supplemental materials. R-package is available at Github repository (https://github.com/zhangwenda1990/integrative).

## Supplementary Information

Below is the link to the electronic supplementary material.

Supplementary file 1 (pdf 645 KB)

## Declarations

### Data Availability

The data that support the findings of this study are openly available in GEO at https://www.ncbi.nlm.nih.gov/geo/query/acc.cgi?acc=GSE110828; accession number is GSE110828.
