# Supplementary material for: Multi-omics Integrative Analysis for Incomplete Data Using Weighted p-Value Adjustment Approaches
Source: J Agric Biol Environ Stat. 2024 Feb 28;30(3):601–17. doi: 10.1007/s13253-024-00603-3 (PMC12274148; doi:10.1007/s13253-024-00603-3)
Supplement: Supplementary file 1 — (pdf 645 KB) [file 13253_2024_603_MOESM1_ESM.pdf]

Supporting Information for "Multi-omics Integrative Analysis for Incomplete Data Using Weighted p-value Adjustment Approaches"

Wenda Zhang[1], Zichen Ma[2], Yen-Yi Ho[3], Shuyi Yang[3], Joshua D. Habiger[4], and Hsin-Hsiung Huang[5]

[1]Walmart Global Tech, Sunnyvale, California 94086, USA

[2]Department of Mathematics, Colgate University, Hamilton, NY 13346, USA

[3]Department of Statistics, University of South Carolina, Columbia, SC 29208, USA

[4]Department of Statistics, Oklahoma State University, Still water, OK 74078, USA

[5]Department of Statistics and Data Science, University of Central Florida, Orlando, FL 32816, USA

Web Appendix A

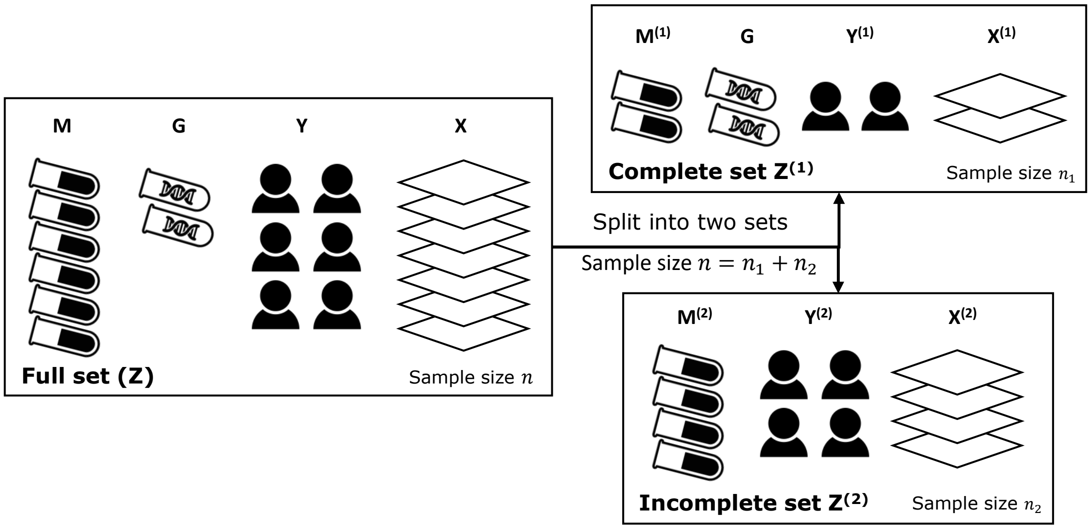

Web Figure 1: Data structure of the full data with complete DNA methylation **M**, covariates **X**, and phenotypic outcome **Y**, and the incomplete gene expression **G**. Note that in some situations, the covariates **X** may not be included in the study.

Web Appendix B

This section provides additional simulation study results in Section 3.

Web Appendix B.1

Web Figure 2 provides the comparisons in statistical power for the proposed omnibus method, the general weighting and the reverse weighting scheme as described in the Section 3.1 in the main article. The DNA-gene association (γ_{MG}) was set equal to 0, 0.1, 0.2, and 0.5; the gene-phenotype association (γ_{GY}) was set to be 0.1 and 0.2; and the missing rate was set to be 70%.

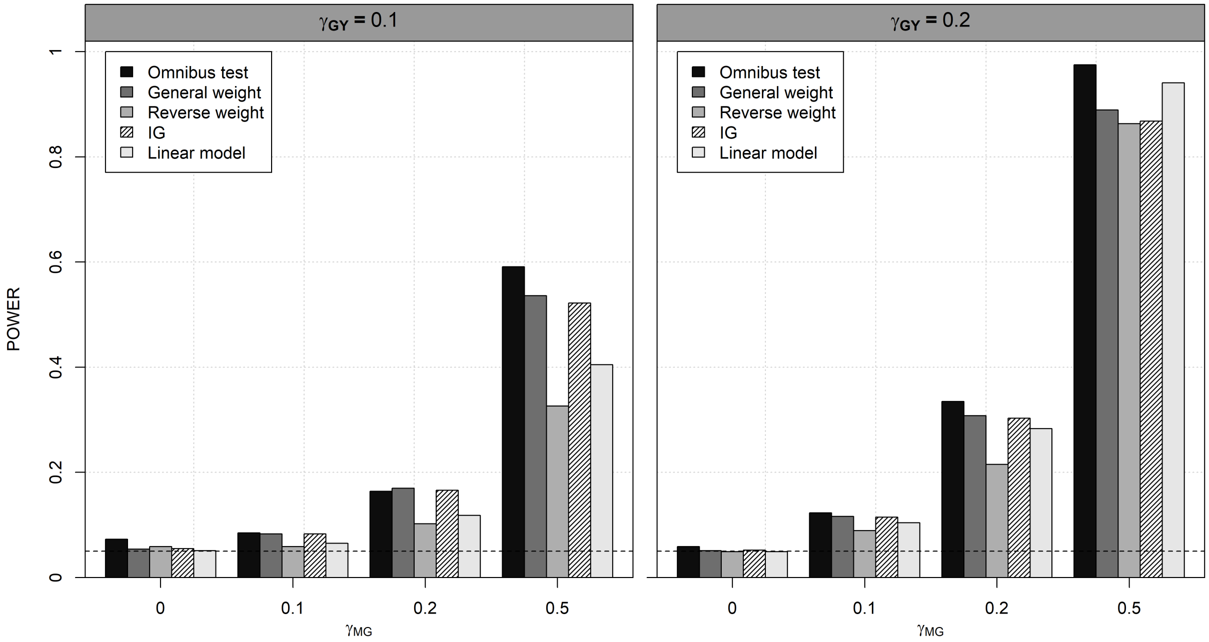

Web Figure 2: Power comparisons of omnibus method, general weight scheme, reverse weight scheme, IG and linear model for various γ_{MG} and γ_{GY} with different missing rates.

Web Appendix B.2

Scenario IV. We conducted another simulation study where $\mathbf{G}_i$ is associated with multiple CpG sites. In detail, we generated $q = 25$ DNA methylation loci independently from a standard normal distribution. The data were simulated by assuming gene expression $\mathbf{G}_i$ is associated with the first five CpG sites:

$$\mathbf{G}_i = \gamma_{0G} + \sum_{j=1}^{5} M_{ij}\gamma_{MG_j} + \mathbf{X}_i^T \gamma_{XG} + \epsilon_{i1}.$$

The regression coefficients γ_{MG_2} to γ_{MG_5} were fixed at 0.5, while γ_{MG_1} (corresponding to the first CpG site) was varied from 0 to 0.5. All other settings, including the magnitude of γ_{GY}, the simulation of covariates $\mathbf{X}$ and the phenotype Y, and the sample size, were all kept the same as in Scenario II. We computed the power of the proposed omnibus method for identifying the underlying CpG site.

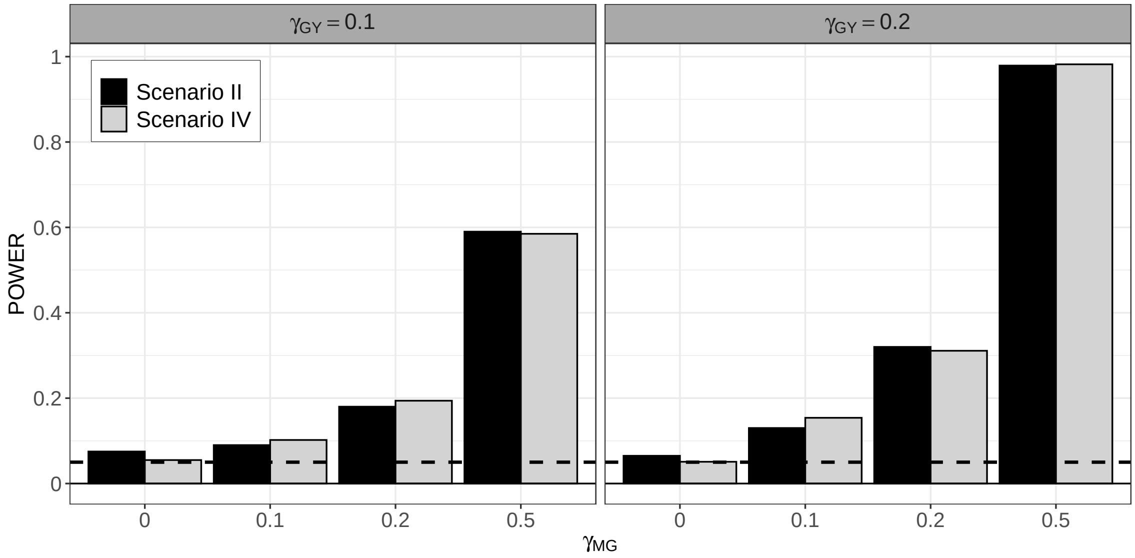

Web Figure 3: Power comparison of the omnibus test in simulation Scenario II and Scenario IV for identifying the significant CpG site. In Scenario II, the phenotype is associated with a single CpG site. In Scenario IV, the phenotype is associated with 5 different CpG sites, and the power is about testing for the association with the first CpG site M_{i1}. Power was calculated over 1000 random samples of size $n = 150$.

Web Figure 3 presents a comparison of the power for identifying the first CpG site between Scenario II and Scenario IV. Overall, there is minimal difference between the two scenarios.

Web Appendix C

In this section, we present additional results for the experimental data analysis. As discussed in Section 4 in the main article, we implemented our proposed omnibus weighting approach to a preterm infants' birth weight dataset described in Section 2 of the main article. The clinical covariates included paternal age, maternal age, paternal body mass index (BMI), maternal BMI, maternal smoking status before pregnancy, the gender of the infants and the missing group which is a binary variable with 0 representing the complete set and 1 denoting the incomplete set.

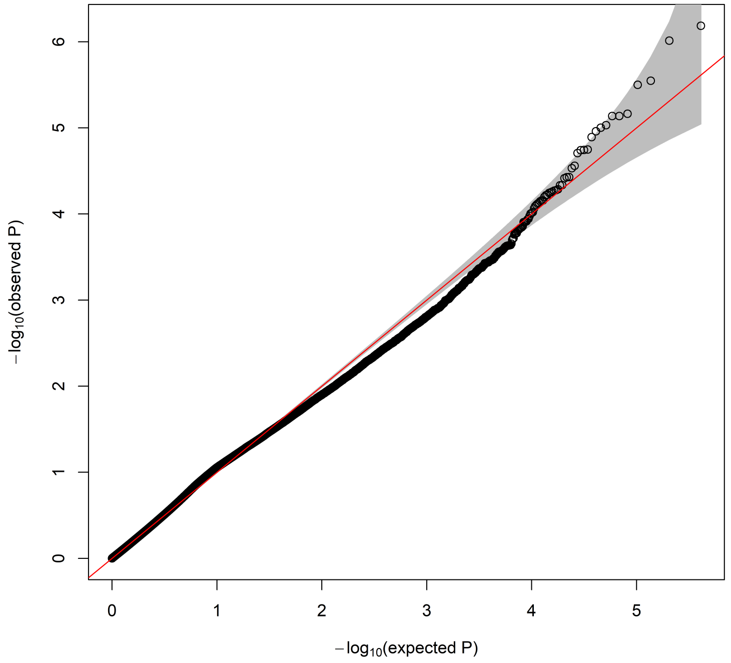

Web Figure 4: Quantile-quantile plot for p-values after SVA and genomic inflation factor adjustment for the proposed omnibus method.

The surrogate variable analysis (SVA) was implemented to account for the unobserved effect of the clinical covariates. After including surrogate variables into the covariate matrix and implementing the models, we used the genomic inflation factor to adjust the p-values. As presented in Web Figure 4, the omnibus method suggests proper type I error control after SVA and genomic inflation factor adjustment.

Web Appendix D

Web Table 1: CpG sites with significant association to birth weight standard deviation in Kashima et al. (2021). Weighted p-values and q-values are calculated based on the proposed method.

	Chromosome	CpG site	Gene	Weighted p-value	q-value
1	6	cg14777908		0.0011	0.6509
2	2	cg12655542		0.0124	0.6509
3	6	cg25683811		0.0233	0.6509
4	7	cg23062810	CLIP2	0.0277	0.6510
5	16	cg06114987	FOXL1	0.0293	0.6511
6	6	cg18520925	C6orf222	0.0325	0.6569
7	3	cg03795847	ZNF167	0.0417	0.6903
8	16	cg03309308		0.0452	0.6994
9	14	cg04407490	JDP2	0.0466	0.7044
10	17	cg21808406	CD68	0.0629	0.7585
11	1	cg07813265	CDC14A	0.0670	0.7713
12	7	cg01049678	GIMAP8	0.0792	0.8014
13	2	cg26204079	DGKD	0.0794	0.8020
14	19	cg07815836	ZNF274	0.0843	0.8126
15	19	cg01453814	SLC6A16	0.0853	0.8150
16	12	cg15085883		0.0899	0.8257
17	11	cg14331899	CYBASC3	0.1008	0.8484
18	17	cg14004678	CANT1	0.1091	0.8629
19	19	cg03722295		0.1131	0.8700
20	22	cg21711214	HPS4	0.1183	0.8778
21	1	cg24955743	PANK4	0.1333	0.8980

Continued on next page

	Chromosome	CpG site	Gene	Weighted p-value	q-value
22	2	cg23925201		0.1529	0.9218
23	2	cg14676702	C2orf77	0.1599	0.9272
24	16	cg10227678	FTO	0.1628	0.9293
25	16	cg08732950	CBFA2T3	0.1659	0.9318
26	1	cg21144009	PLXNA2	0.1721	0.9363
27	11	cg07441944	CD82	0.1890	0.9470
28	12	cg02742418	SLC15A4	0.1894	0.9471
29	1	cg26266618	CD34	0.1920	0.9491
30	1	cg10495683	BCAR3	0.1939	0.9507
31	8	cg13394182	TOP1MT	0.2080	0.9577
32	1	cg15497761	TRIM46	0.2198	0.9636
33	8	cg02007288	GTF2E2	0.2225	0.9647
34	3	cg25773386	GPR62	0.2249	0.9659
35	6	cg08200851	FARS2	0.2280	0.9674
36	6	cg05094429	CCR6	0.2283	0.9677
37	5	cg21869586		0.2360	0.9701
38	3	cg13586051	DNASE1L3	0.2374	0.9706
39	1	cg10965178	TIE1	0.2386	0.9709
40	16	cg00396427	ARHGAP17	0.2560	0.9756
41	1	cg24422489	FCGR2A	0.2767	0.9790
42	7	cg19629631	MAD1L1	0.2853	0.9804
43	12	cg00159243	SELPLG	0.2916	0.9815
44	6	cg25325512	PIM1	0.2919	0.9816
45	4	cg10868668		0.2996	0.9826
46	5	cg01843999		0.3146	0.9854

Continued on next page

	Chromosome	CpG site	Gene	Weighted p-value	q-value
47	11	cg11328127	SIGIRR	0.3158	0.9854
48	15	cg01959287		0.3161	0.9854
49	12	cg21249754	PTPN6	0.3251	0.9874
50	7	cg15031685	FLNC	0.3448	0.9892
51	11	cg25068347	ETS1	0.3557	0.9911
52	2	cg07185119	EHD3	0.3558	0.9912
53	16	cg09504873	DYNC1LI2	0.3581	0.9913
54	6	cg16199747		0.3586	0.9913
55	15	cg13206220	RGMA	0.3732	0.9927
56	4	cg05205842	KLF3	0.3773	0.9930
57	10	cg20197123		0.3839	0.9937
58	1	cg21932672	TMEM56	0.3881	0.9940
59	1	cg25317315	PDE4DIP	0.3884	0.9940
60	2	cg11708690		0.3984	0.9953
61	20	cg11284582		0.4007	0.9953
62	19	cg27064845	GLTSCR2	0.4129	0.9954
63	1	cg05034603	SLC2A1	0.4142	0.9954
64	10	cg24110396	CCNY	0.4154	0.9954
65	6	cg07507418	TRERF1	0.4211	0.9954
66	17	cg04055490		0.4260	0.9954
67	16	cg26947831		0.4487	0.9954
68	16	cg27519828		0.4587	0.9954
69	17	cg17588003	C17orf87	0.4610	0.9956
70	12	cg17277939	BEST3	0.4766	0.9965
71	13	cg06329392		0.4916	0.9970

Continued on next page

	Chromosome	CpG site	Gene	Weighted p-value	q-value
72	22	cg04630823	NUP50	0.5011	0.9970
73	15	cg02964434		0.5023	0.9970
74	1	cg07996594	RUNX3	0.5149	0.9970
75	19	cg07627628	DNMT1	0.5209	0.9970
76	1	cg01949002	SKI	0.5331	0.9970
77	1	cg12126344		0.5363	0.9970
78	10	cg07904073		0.5491	0.9970
79	3	cg11291773		0.5527	0.9970
80	1	cg17461600	DAB1	0.5657	0.9970
81	1	cg02896872	ITPKB	0.5725	0.9970
82	11	cg01471232	SIGIRR	0.5966	0.9970
83	18	cg12182124	LAMA3	0.5972	0.9970
84	15	cg17232357	SMAD6	0.5994	0.9970
85	19	cg00041047	SLC6A16	0.6036	0.9970
86	1	cg12467404	CD55	0.6105	0.9970
87	7	cg16356456	CLIP2	0.6123	0.9970
88	10	cg00602416		0.6131	0.9970
89	2	cg08239893		0.6159	0.9970
90	15	cg06797389	PATL2	0.6184	0.9970
91	4	cg22778014	COL25A1	0.6226	0.9970
92	16	cg04400455		0.6416	0.9970
93	10	cg16704246	RBM20	0.6423	0.9970
94	2	cg19744173	FBLN7	0.6489	0.9970
95	16	cg01445100	BANP	0.6546	0.9970
96	12	cg24032056		0.6604	0.9970

Continued on next page

	Chromosome	CpG site	Gene	Weighted p-value	q-value
97	5	cg07340025		0.6734	0.9970
98	17	cg09121543	LIMD2	0.6914	0.9970
99	6	cg01066157	SYNE1	0.6931	0.9970
100	7	cg06567596	LMTK2	0.6976	0.9970
101	10	cg20125920	C10orf140	0.7066	0.9970
102	14	cg14412134	MTHFD1	0.7079	0.9970
103	1	cg17855595	ZNF692	0.7091	0.9970
104	18	cg16113793	LAMA3	0.7185	0.9970
105	2	cg17501982	NCK2	0.7363	0.9970
106	17	cg22093121	ABCA9	0.7450	0.9970
107	1	cg19266329		0.7460	0.9970
108	10	cg01861509	SPOCK2	0.7510	0.9970
109	11	cg03169242		0.7595	0.9970
110	11	cg01218206	SIK3	0.7716	0.9970
111	7	cg00376553	TSC22D4	0.7724	0.9970
112	13	cg13121938	RASA3	0.7856	0.9970
113	10	cg05135499		0.7964	0.9970
114	5	cg03605542	DEPDC1B	0.7998	0.9970
115	11	cg20363347	MYEOV	0.8232	0.9970
116	11	cg04608933	KCNQ1	0.8315	0.9970
117	2	cg04458969		0.8350	0.9970
118	16	cg16727774	CA5A	0.8369	0.9970
119	1	cg27333706	LOC642587	0.8538	0.9970
120	2	cg18866212		0.8570	0.9970
121	19	cg06237487	SLC6A16	0.8707	0.9970

Continued on next page

	Chromosome	CpG site	Gene	Weighted p-value	q-value
122	7	cg23122899	WIPI2	0.8865	0.9970
123	1	cg03731202		0.8871	0.9970
124	8	cg00345025		0.8885	0.9970
125	7	cg26959945	MACC1	0.8927	0.9970
126	10	cg19791727		0.8981	0.9970
127	9	cg27592318	HEMGN	0.9013	0.9970
128	7	cg02944312		0.9017	0.9970
129	14	cg00554993	CEBPE	0.9174	0.9970
130	3	cg13793580	LRRIQ4	0.9310	0.9970
131	13	cg21565496		0.9320	0.9970
132	3	cg21585138	CISH	0.9328	0.9970
133	22	cg07680505		0.9334	0.9970
134	18	cg15871086		0.9412	0.9970
135	3	cg09866743	ARPP-21	0.9415	0.9970
136	12	cg04456029	DTX1	0.9508	0.9970
137	22	cg21564495	FAM19A5	0.9610	0.9970
138	22	cg02532700	NCF4	0.9620	0.9970
139	4	cg21542842	11-Sep	0.9644	0.9970
140	12	cg18173184		0.9645	0.9970
141	22	cg05546044	MAPK1	0.9671	0.9970
142	8	cg23822276	C8orf79	0.9677	0.9970
143	19	cg26257814	FLJ26850	0.9710	0.9970
144	10	cg18515031	C10orf104	0.9714	0.9970
145	11	cg00233028		0.9738	0.9971
146	1	cg11134246	CCDC24	0.9863	0.9975

Continued on next page

	Chromosome	CpG site	Gene	Weighted p-value	q-value
147	6	cg26870746	TAGAP	0.9868	0.9975
148	6	cg01340991	SYNGAP1	0.9874	0.9975
149	13	cg17061183	RASA3	0.9934	0.9977
150	7	cg09282258	PRR15	0.9935	0.9977

References

Kashima, K., Kawai, T., Nishimura, R., Shiwa, Y., Urayama, K. Y., Kamura, H., Takeda, K., Aoto, S., Ito, A., Matsubara, K., et al. (2021). Identification of epigeneticsic memory candidates associated with gestational age at birth through analysis of methylome and transcriptional data. *Scientific reports*, 11(1):1–16.
